# Evidence for OTUD-6B Participation in B Lymphocytes Cell Cycle after Cytokine Stimulation

**DOI:** 10.1371/journal.pone.0014514

**Published:** 2011-01-18

**Authors:** Zhongping Xu, Yufang Zheng, Yufei Zhu, Xiangyin Kong, Landian Hu

**Affiliations:** 1 The Key Laboratory of Stem Cell Biology, Institute of Health Sciences, Shanghai Institutes for Biological Sciences (SIBS), Chinese Academy of Sciences (CAS) and Shanghai Jiao Tong University School of Medicine (SJTUSM), Shanghai, People's Republic of China; 2 Department of Physiology and Biophysics, School of Life Sciences, Fudan University, Shanghai, People's Republic of China; 3 State Key Laboratory of Medical Genomics, Ruijin Hospital, Shanghai Jiaotong University, Shanghai, People's Republic of China; 4 Graduate School of the Chinese Academy of Sciences, Beijing, People's Republic of China; The Salk Institute, United States of America

## Abstract

Deubiquitinating enzymes (DUBs) are important regulators of cell proliferation. Here we identified a functional deubiquitinating enzyme, ovarian tumor domain-containing 6B (OTUD-6B). Mutation of the conserved Cys residue abolished its deubiquitinating activity *in vitro*. Otud-6b expression was induced with cytokine stimulation in both mouse Ba/F3 cells and primary B lymphocytes followed a rapid decrease. This rapid decrease was partially facilitated by tristetraprolin (TTP) destabilization of Otud-6b mRNA through AU-rich motifs. Enforced expression of OTUD-6B in Ba/F3 cells could block cell proliferation by arresting cells in G1 phase. In addition, cyclin D2 level was down-regulated when OTUD-6B WT was overexpressed. Therefore, down-regulation of Otud-6b expression after prolonged cytokine stimulation may be required for cell proliferation in B lymphocytes.

## Introduction

The ubiquitin-mediated proteolytic pathway is involved in multiple cellular processes including cell cycle regulation [1], transcriptional activation [2], and antigen presentation [3]. In addition to ubiquitination, the importance of deubiquitinating enzymes (DUBs) has been demonstrated recently [4], [5], [6]. DUBs can either recycle ubiquitin as components of the 26S proteasome [7] or rescue proteins from the degradation pathway by deubiquitination [7], [8]. There are five sub-families of DUBs classified by their sequence diversity: the ubiquitin C-terminal hydrolases (UCHs), the ubiquitin-specific peptidases (USPs/UBPs), the ovarian tumor (OTU) domain proteins, the Josephin or Machado-Joseph disease (MJD) proteins, and the JAMM (Jab1/MPN domain-associated metalloisopeptidase) domain proteins. The JAMM proteins are zinc metalloisopeptidases, while the other four families are cysteine peptidases [9].

B cell fate is essentially associated with the adaptive immune system [10], and B cell fate is modulated by cytokines during its maturation, homeostasis, and proliferation through target genes expression [11], [12]. Recently, mouse Dub-1, Dub-1a, Dub-2, and Dub-2a were reported to be hematopoietic-specific DUBs in B lymphocytes. Their expression levels were rapidly induced upon cytokine stimulation, which is probably due to the cytokine-inducible enhancer in the 5′-UTR [13], [14]. Interestingly, the expression levels of those DUBs were sharply down-regulated following the fast induction and little is known about this fast down-regulation. These four DUBs belong to the USP17 gene family, members of which form part of highly polymorphic tandem repeat sequences on mouse chromosome 7 [15]. There is no other DUB reported to present this induction-decline expression pattern. More recently, microarray data have shown that many OTU family members were rapidly up-regulated or down-regulated in human esophageal epithelial cells and lymphocytes when stimulated by different cytokines, such as ovarian tumor domain containing 6B (OTUD-6B), a novel DUB of the OTU family members. OTUD-6B was originally named as CGI-77 and has deubiquitinating enzyme activity *in vitro* [16]. It was up-regulated on human esophageal epithelial cells after interleukin-13 (IL-13) stimulation [17]. BAFF, a B cell-activating factor of the TNF family, could also induce Otud-6b expression on mouse B cells after 4 hours stimulation [18]. However, granulocyte colony-stimulating factor (G-CSF) could effectively down-regulate OTUD-6B expression when human leukocytes were stimulated for 16 hours [19]. Although these experiments showed that OTU family members were regulated by cytokines, little is known about the mechanism and function of such regulations.

Here we report that Otud-6b, a functional DUB of the OTU family, can be induced by IL-3, IL-4, IL-13 and granulocyte-macrophage colony-stimulating factor (GM-CSF) stimulation in B lymphocytes. However, prolonged stimulation with these cytokines effectively decreased the expression of Otud-6b. This is the first OTU family member to be reported to have such cytokines response. To further investigate the down-regulation mechanism, we knocked down several proteins involved in mRNA regulation and found that tristetraprolin (TTP) was responsible for Otud-6b mRNA rapid degradation. Enforced expression of OTUD-6B could block cell growth and arrest cells in G1 phase. Apoptosis assays showed that overexpression of OTUD-6B in Ba/F3 cells increased the number of cells in subG1 and pro-apoptotic stages. In addition, cyclin D2 expression level was down-regulated when OTUD-6B WT was overexpressed in Hela and Ba/F3 cells, while overexpression of OTUD-6B C188S, which abolished its deubiquitinating activity, had no effect on cyclin D2 level. Therefore, OTUD-6B may participate in cell cycle regulation in B lymphocytes after cytokine stimulation.

## Results

### OTUD-6B is a Functional Deubiquitinating Enzyme

**Figure 1:**
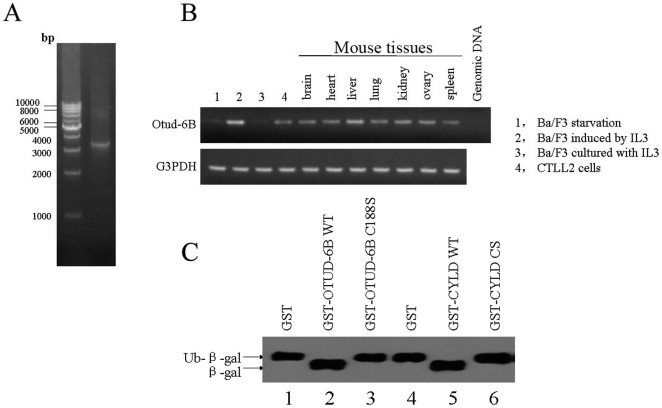
OTUD-6B is a functional deubiquitinating enzyme. **A.** Molecular cloning of the cDNA of human OTUD-6B was amplified from total RNA of Raji cells and subjected to 1% agarose gel analysis. **B.** The expression of mouse Otud-6b mRNA in Ba/F3 cells [5], CTLL-2 cells and various tissues (brain, heart, liver, lung, kidney, ovary, and spleen) was analyzed by RT-PCR using Otud-6b-specific primers. G3PDH was used as control. Ba/F3 cells were kindly supplied by prof. Xin yuan Liu (Shanghai Institute of Biochemistry and Cell Biology, SIBS, CAS). CTLL2 cells were provided by the Cell Bank, Shanghai Institute of Biochemistry and Cell Biology, SIBS, CAS. **C.** Ub-Met-β-gal fusion protein was prepared from MC1061 cells. The supernatant was incubated with purified GST (lane 1), OTUD-6B WT (lane 2), OTUD-6B C188S (lane 3), GST (lane 4), GST-CYLD WT (lane 5), and GST-CYLD CS (lane 6) fusion protein at 4°C with rotation for 4 hours. Both OTUD-6B WT and GST-CYLD WT could cleave ubiquitin from the Ub-Met-β-gal fusion protein.

Human OTUD-6B, also named as DUBA5 and CGI-77, is located on Chr8: 92151719-92168498 [20]. Specific primers were designed to amplify OTUD-6B cDNA from Raji cells by RT-PCR (**Figure 1A**). The sequence of OTUD-6B cDNA clone was identical to GeneBank NM_016023. The full-length OTUD-6B cDNA is 3306 bp and contains a 972-bp ORF. The mouse homolog Otud-6b cDNA is 3311 bp long and consists of seven exons encoding a 325-amino acid mouse Otud-6b protein. The protein homology between human OTUD-6B and mouse Otud-6b is about 87% (**Figure S1**). We analyzed the expression pattern of Otud-6b mRNA in mouse tissues by RT-PCR using the Otud-6b specific primers. RT-PCR results revealed that Otud-6b mRNA is expressed in various mouse tissues, including brain, heart, lung, kidney, ovary, spleen, and B lymphocytes (**Figure 1B**), which indicated that Otud-6b is probably a widely expressed housekeeping gene.

Next we investigated whether OTUD-6B is a functional deubiquitinating enzyme. Sequence alignment on human OTU family members indicated that the Cys_188_ is the putative conserved Cys residue in OTUD-6B [21]. Therefore, we mutated this site into a Ser to generate an OTUD-6B C188S mutant. *In vitro* deubiquitinating enzyme assay showed that GST-OTUD-6B WT fusion protein could deubiquitinate Ub-Met-β-gal to an extent comparable to GST-CYLD, which is a reported functional DUB [22], [23], while the OTUD-6B C188S mutant failed to cleave the Ub-Met-β-gal substrate (**Figure 1C**). Immunoblot confirmed that all GST fusion proteins were synthesized effectively (**Figure S2**). These results demonstrated that OTUD-6B is a functional deubiquitinating enzyme *in vitro*.

### Cytokines could Induce Otud-6b Expression in B lymphocytes Followed by a Rapid Decline

**Figure 2:**
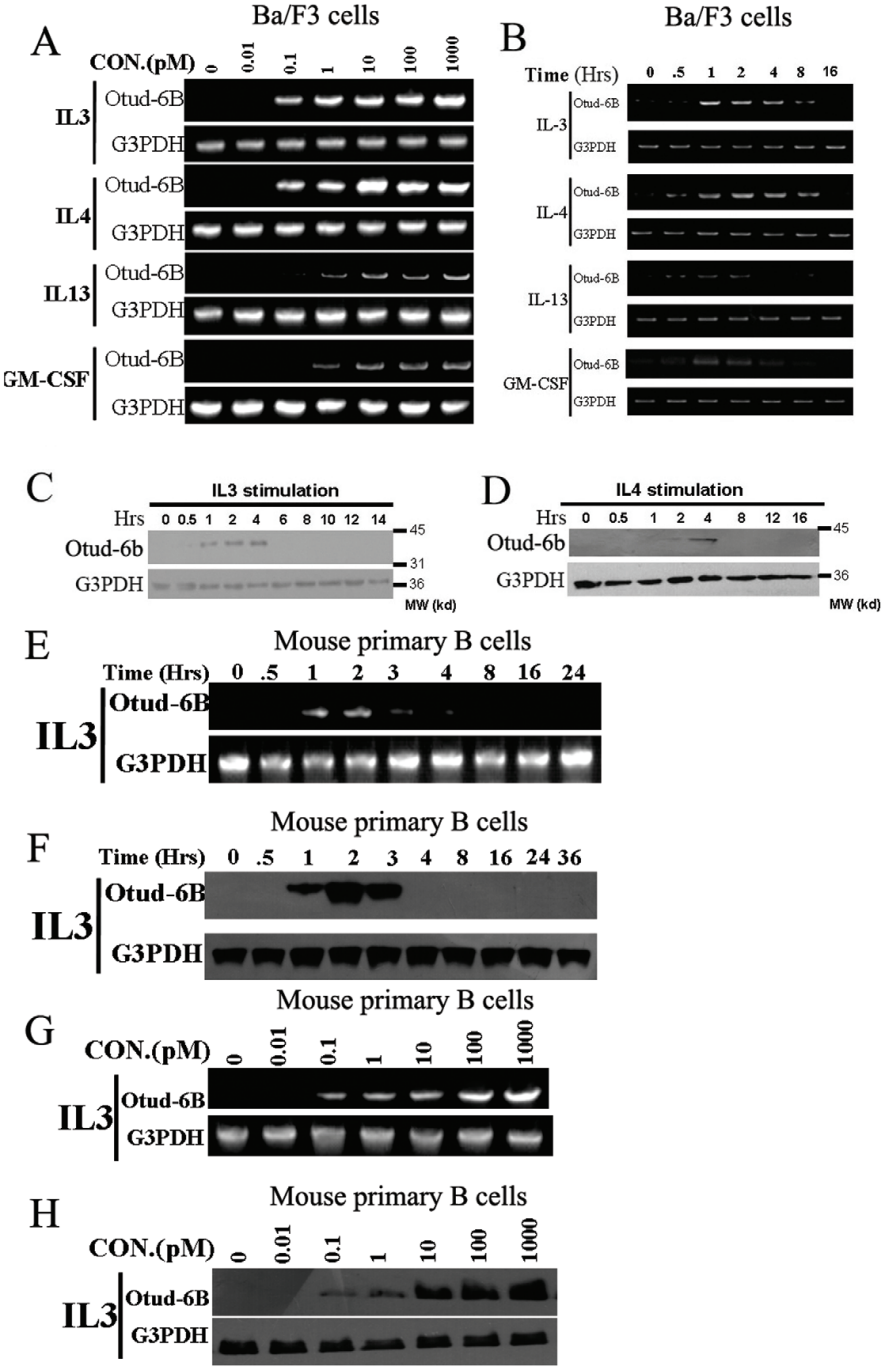
Otud-6b expression is induced by cytokines following by a rapid decline. **A.** Otud-6b RNA level in Ba/F3 cells after starvation and two hours stimulation with different concentrations (0, 0.01, 0.1, 1, 10, 100, and 1000 pM) of mouse IL-3, IL-4, IL-13, and GM-CSF. **B.** Otud-6b RNA level in Ba/F3 cells after starvation and stimulation with 10 pM mouse IL-3, IL-4, IL-13, and GM-CSF for the indicated times (0, 0.5, 1, 2, 4, 8, and 16 hours). **C&D.** Ba/F3 cells were starved and stimulated with 10 pM mouse IL-3 or IL-4 for the indicated time. Otud-6b expression of the Ba/F3 cell lysates was immunoblotted using anti-OTUD-6B antibody. G3PDH was used as a loading control. **E&F.** Otud-6b RNA level and protein level in primary mouse B cells after starvation and stimulation with 10 pM mouse IL-3 for the indicated times (0, 0.5, 1, 2, 3, 4, 8, 16, and 24 hours). G3PDH was used as a loading control. **G&H.** Otud-6b RNA level in primary mouse B cells after starvation and two hours stimulation with different concentrations (0, 0.01, 0.1, 1, 10, 100, and 1000 pM) of mouse IL-3. G3PDH was used as a loading control.

As microarray data have showed that OTUD-6B expression levels could be regulated upon cytokine stimulation [17], [18], [19], [20]. To investigate the response of Otud-6b expression levels to cytokine stimulation in B lymphocytes, we first examined that in Ba/F3 cells, a mouse pro-B cell line. The mRNA levels of Otud-6b showed a dose-dependent response after 2 hours incubation with different concentrations of IL-3, IL-4, IL-13, and GM-CSF (0, 0.01, 0.1, 1, 10, 100, and 1000 pM) (**Figure 2A**). We also tested the time course response for Otud-6b mRNA expression in Ba/F3 cells under 10 pM IL-3, IL-4, IL-13, or GM-CSF stimulation (**Figure 2B**). Our results showed that Otud-6b mRNA expression levels were increased from 0 to 2 hours but decreased rapidly after 4–6 hours with those cytokines stimulation. On the other hand, IL-2 could not induce Otud-6b expression in Ba/F3 cells (**Figure S3**). A rabbit polyclonal antibody for Otud-6b/OTUD-6B was developed to facilitate our studies on the endogenous protein (**Figure S4**). Then endogenous Otud-6b expression changes were tested in similar time course experiments with 10 pM mouse IL-3 or IL-4. Immunoblot results showed that endogenous Otud-6b protein was induced after 1 hour IL-3 stimulation and declined after 4 hours (**Figure 2C**), while the response with IL-4 stimulation was slower as endogenous Otud-6b protein expression could only be detected after 4 hours and declined after 6 hours of stimulation (**Figure 2D**). Such difference on mRNA kinetics between IL-3 and IL-4 stimulation is probably due to different downstream signaling pathways induced by those two cytokines [24].

To confirm the induction effect of Otud-6b in B lymphocytes, we next conducted similar experiments in primary B cells from C57BL/6 mice. Similar time course and concentration course experiments were performed on those primary B cells with IL-3 stimulation. The time course experiments showed that both Otud-6b mRNA and protein were induced after 1 hours' stimulation and declined after 3 hours' stimulation (**Figure 2E&F**). The concentration course experiments showed that the induced Otud-6b mRNA and protein could be detected with 0.1 pM or higher IL-3 stimulation for 2 hours (**Figure 2G&H**). Therefore, Otud-6b could also be induced in mouse primary B cells with IL-3 stimulation. Although this cytokine stimulation pattern has been reported for Dub-1 and Dub-2a, Otud-6b is the first OTU family member found to be regulated by cytokines in B lymphocytes.

### OTUD-6B Overexpression Slows Proliferation and Increases the Rate of Apoptosis

**Figure 3:**
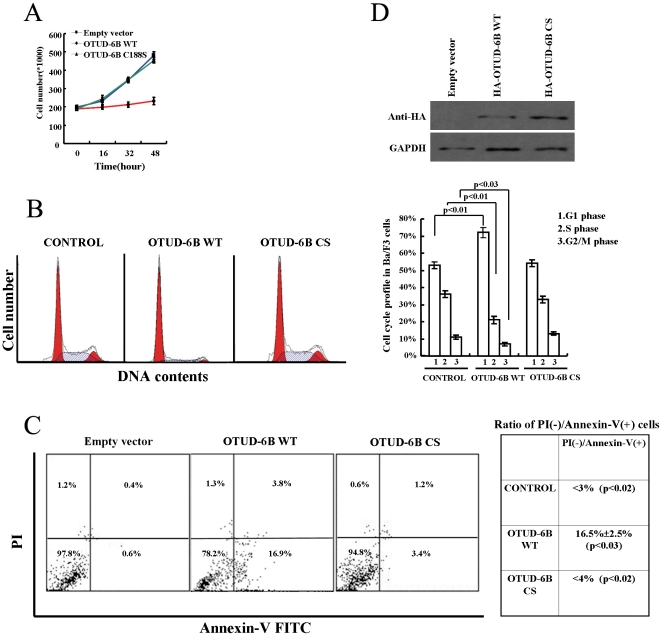
OTUD-6B overexpression slows proliferation and increases the rate of apoptosis. **A.** Ba/F3 cells were transfected with pcDNA3.1(+), OTUD-6B WT, and OTUD-6B C188S vectors. Thirty-two hours later, Ba/F3 cells were seeded at 2×10^5^ cells per ml and analyzed at 16-hour intervals by trypan blue staining. **B.** Ba/F3 cells were transfected with pcDNA3.1(+), OTUD-6B WT, and OTUD-6B C188S vectors. Thirty-two hours later, cells were stained using PI and analyzed by flow cytometry. Cell cycle profile from three independent experiments was calculated statistically. **C.** PI-Annexin V analysis among pcDNA3.1(+), OTUD-6B WT, and OTUD-6B C188S-expressing Ba/F3 cells. All experiments were repeated three times independently. **D.** Ba/F3 cells were transfected with pcDNA3.1(+), OTUD-6B WT, and OTUD-6B C188S vectors. Thirty-two hours later, cell extracts were analyzed by immunoblot with anti-HA antibody. GAPDH was used as a loading control.

During our cytokine stimulation experiments, we observed an interesting rapid down-regulation of Otud-6b after prolonged cytokine stimulation. We wanted to understand why Otud-6b is so rapidly down-regulated after prolonged stimulation. Therefore, we enforced OTUD-6B expression in Ba/F3 cells to overturn the down-regulation. Interestingly, overexpression of OTUD-6B could affect cell proliferation and cell cycle in Ba/F3 cells. While cells transfected with pcDNA3.1(+)and OTUD-6B C188S mutant vector doubled 2-3 times after 48 hours of culture, OTUD-6B WT vector transfected cells showed a substantial reduction in the proliferation rate (**Figure 3A**). PI staining was also performed on those cells. There were only about 28% OTUD-6B WT transfected cells in S and G2/M phase while there were 47% control cells and 46% OTUD-6B C188S transfected cells in those phases (**Figure 3B**). Therefore, there were more OTUD-6B WT transfected cells arrested in G1 phase (72% versus 53% in control and 54% in OTUD-6B C188S transfected cells, **Figure 3B**). Moreover, PI-Annexin V assays were also performed on Ba/F3 cells 32 hours after transfection. The ratio of PI^(−)^/AnnexinV^(+)^ cells in OTUD-6B WT expressing cells was 16.5%±2.5% (p<0.03), which was significantly higher than that of pcDNA3.1(+) vector and OTUD-6B C188S transfected cells (**Figure 3C**) even though the expression levels of wild-type and mutant OTUD-6B were similar (**Figure 3D**). These findings indicated that overexpression of OTUD-6B WT in Ba/F3 cells can block cell proliferation and lead to apoptosis, while down-regulation of Otud-6b in Ba/F3 cells has no such effect (**Figure S5**).

### Cyclin D2 was Down-regulated when OTUD-6B was Overexpressed

**Figure 4:**
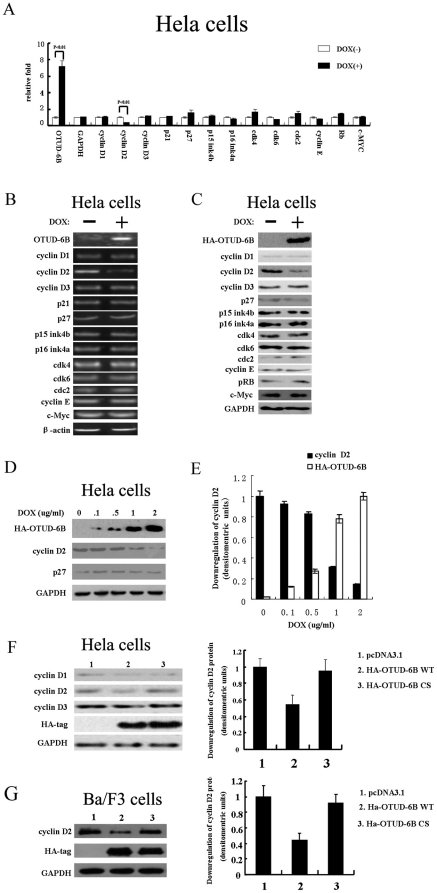
Cyclin D2 level is down-regulated with OTUD-6B overexpression. **A & B & C.** Real-time PCR, RT-PCR, and immunoblot of OTUD-6B, GAPDH, cyclin D1, cyclin D2, cyclin D3, p21, p27, p15, p16, cdk4, cdk6, cdc2, cyclin E, Rb, and c-Myc genes from total RNA samples and protein extracts of DOX(−) and DOX(+) Hela cells. Hela cells were provided by the Cell Bank, Shanghai Institute of Biochemistry and Cell Biology, SIBS, CAS. **D & E.** Immunoblot of OTUD-6B, cyclin D2, and p27 with gradient concentrations (0, 0.1, 0.5, 1, 2 ug/ml) of DOX stimulation. Quantitation was performed with Bandscan 5.0 from 3 independent results. **F & G.** Immunoblot (left panel) for cyclin D1, cyclin D2, and cyclin D3 on Hela and Ba/F3 cells overexpressing pcDNA3.1(+), HA-OTUD-6B WT, and HA-OTUD-6B C188S. Quantitation (right panel) of cyclin D1, cyclin D2, and cyclin D3 on Hela and Ba/F3 cells was performed with Bandscan 5.0 from three independent results.

Next we checked the cell cycle regulator(s) in OTUD-6B expressing cells. Real-time PCR of the cell cycle regulators was performed on the RNAs from Tet-On advanced HA-OTUD-6B Hela cells with or without DOX induction. OTUD-6B mRNA level in DOX(+) cells was about 7 times higher than that in DOX(−) cells and cyclin D2, a G1/S cell cycle regulator, was down-regulated about 70% in DOX(+) cells compared to DOX(−) cells (**Figure 4A**). There is no significant difference on the mRNA levels of the other regulators (cyclin D1, cyclin D3, p21, p27, p15, p16, cdk4, cdk6, cdc2, cyclin E, Rb, and c-Myc genes). We also confirmed this down-regulation of cyclin D2 through RT-PCR and immunoblot (**Figure 4B** **&** **Figure 4C**). Moreover, down-regulation of cyclin D2 was correlated with increased HA-OTUD-6B WT expression (**Figure 4D** **&** **Figure 4E**). OTUD-6B C188S had no effect on cyclin D2 (**Figure 4F**). Similar results were also obtained in Ba/F3 cells (**Figure 4G**). Over all, these data indicated that cyclin D2 level is down-regulated in OTUD-6B overexpressing cells and such down-regulation is dependent on OTUD-6B's catalytic activity even though we did not observe any deubiquitinating activity of OTUD-6B on cyclin D2 (data not shown). Down-regulation of OTUD-6B also has no effect on cyclin D2 level in those cells (**Figure S6**).

### TTP Destabilizes Otud-6b mRNA through its 3′UTR

**Figure 5:**
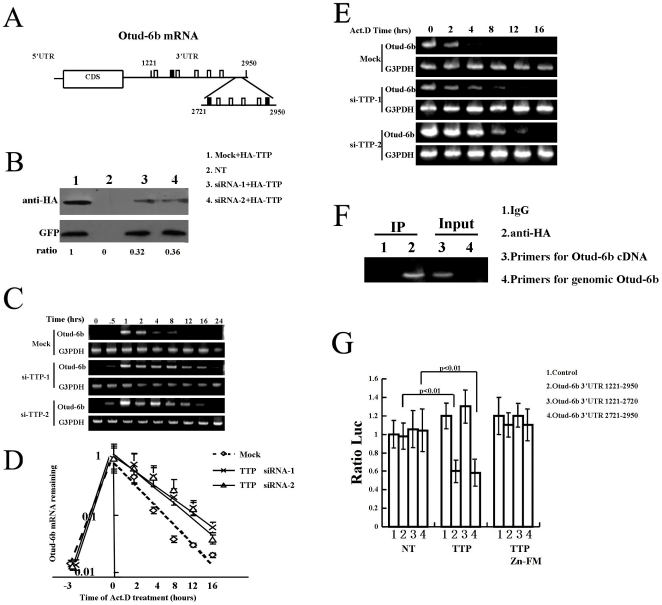
TTP is involved in Otud-6b mRNA rapid degradation after induction. **A.** The sequence of Otud-6b 3′UTR. AUUUA pentamer or AUUUUA hexamer are underlined. Each rectangle indicates an AU-rich sequence site. The AU-rich sequence with U surrounding it is indicated as a black rectangle. **B.** pSUPER Mock, pSUPER siTTP-1 or 2 and HA-TTP vectors co-expressing Ba/F3 cells were seeded at 2×10^5^ cells per ml. Cell extracts were immunoblotted with anti-HA antibody. GFP was used as a loading control. **C.** Otud-6b RNA level in Ba/F3 cells transfected with pSUPER siTTP-1,2 vectors after starvation and stimulation with 10 pM mouse IL-3 for the indicated times (0, 0.5, 1, 2, 4, 8, 12, 16, and 24 hours). **D.** Ba/F3 cells were transfected with pSUPER Mock, pSUPER siTTP-1 or 2 vectors. 24 later, cells were stimulated by 10 pM IL-3 and incubated with 15 ug/ml Act.D for the time indicated. The qRT-PCR was performed to detect Otud-6b mRNA remaining at each time point. **E.** Semi-quantitative RT-PCR analysis of Otud-6b mRNA remaining at each time point after Act.D adding. **F.** RT-PCR for Otud-6b mRNA in protein-mRNA immunoprecipitation complex. The mRNAs of Ba/F3 cells transfected with HA-TTP when response to IL-3 was immunoprecipitated with anti-HA and IgG serum. As a control, Input (10%) denoted that the RNA sample contained Otud-6b mRNA and didn't contain genomic DNA. **G.** TTP transfection had little effect on pGL3 control luciferase expression. Wild-type TTP transfection resulted in a significant decrease in full-length Otud-6b 3′-UTR luciferase reporter expression in 293T cells, while the TTP Zn-FM did not alter Otud-6b 3′-UTR-mediated expression. Different region of Otud-6b 3′UTR was inserted into downstream of luciferase coding region. The truncate 3′UTR vectors were constructed depending on the AU-rich sequence distribution. 293T cells were provided by the Cell Bank, Shanghai Institute of Biochemistry and Cell Biology, SIBS, CAS.

Otud-6b mRNA was rapidly down-regulated after prolonged cytokine induction. In order to investigate the mechanism of such rapid decay, we investigated several possible regulation pathways for mRNA degradation. MicroRNAs are reported to be involved in directing mRNA degradation of the target genes [25]. Down-regulation of Dicer1, which is essential in processing small RNAs, had no effect on Otud-6b mRNA level (**Figure S7**). This data indicated that microRNAs probably do not regulate Otud-6b mRNA stability under our conditions. Several RNA binding proteins including TTP, AU-rich element RNA-binding protein 1 (AUF1), and butyrate response factor-1 (BRF1) have been shown to be able to regulate ARE-mRNAs stability [26], [27], [28]. Therefore, we analyzed Otud-6b mRNA sequence and found that there are several AU-rich sequence motifs in the 3′UTR of mouse Otud-6b (**Figure 5A**). We then checked Otud-6b mRNA levels in Ba/F3 cells when TTP, AUF1, and BRF1 were knocked down respectively. While there was no effect in Otud-6b mRNA levels with AUF1 and BRF1 knock-down (**Figure S7**), the degradation of Otud-6b mRNA was delayed when TTP was effectively down-regulated by pSUPER siTTP-1 and siTTP-2 (**Figure 5B** **&** **Figure 5C**). Both scramble siRNA and mock vector control had no effect on Otud-6b mRNA degradation rate (**Figure S8**). To quantitatively determine the effect of TTP on endogenous Otud-6b mRNA stability, actinomycin D (a transcription inhibitor) chase were performed on Ba/F3 cells treated with IL-3 for 3 hours. The stability of Otud-6b mRNA was significantly increased when TTP was knocked down (t_1/2_ = 2.2±0.2 h (p<0.01) for Mock versus t_1/2_ = 5.6±0.3 h (p<0.02) for siRNA-1 and t_1/2_ = 4.9±0.3 h (p<0.02) for siRNA-2, **Figure 5D**). Semi-quantitative RT-PCR analysis confirmed that Otud-6b mRNA was more stable after TTP knockdown (**Figure 5E**). These data suggest that TTP controls the decay rate of Otud-6b mRNA in response to IL-3 stimulation.

In order to confirm TTP could regulate Otud-6b mRNA, we investigated the specifity and interaction of TTP with Otud-6b mRNA in Ba/F3 cells. We first performed protein-mRNA complex immunoprecipitation assay on HA-TTP expressing Ba/F3 cells. Otud-6b mRNA could be detected by RT-PCR from TTP-RNA complex immunoprecipitated with anti-HA antibody (**Figure 5F**). This data confirmed that TTP could bind to Otud-6b mRNA. To investigate which AU-rich elements on the 3′-UTR of Otud-6b mRNA is involved in TTP regulation, we next constructed a series luciferase reporter with different Otud-6b-specific 3′-UTR regions (1221–2950, 1221–2720, and 2721–2950 of mouse Otud-6b cDNA). As 293T cells have very low endogenous TTP expression [29], we co-transfected HA-TTP and those luciferase reporter constructs into 293T cells. Luciferase expression with Otud-6b 3′-UTR (1221–2950) was significantly inhibited by co-transfection of wild-type TTP in 293T cells, while TTP Zn-FM (TTP zinc finger mutant), which lacks the ability to bind to mRNA, had no effect (**Figure 5G**). These data indicated that the 3′UTR of Otud-6b contributes greatly to the mRNA stability. Furthermore, truncation of Otud-6b 3′-UTR showed that TTP probably binds to the end of Otud-6b 3′-UTR as TTP could significantly reduce luciferase production with the 3′UTR (2721–2950) and has no effect on luciferase expression with the 3′-UTR (1221–2720) (**Figure 5G**). These results indicated the AU-rich sequences in the 3′UTR 2721–2950 region may contain the AU-rich element (ARE), which is responsible for Otud-6b mRNA instability.

## Discussion

In this study, we firstly identified an OTU family member, Otud-6b, to be a functional deubiquitinating enzyme. After serum starvation, Otud-6b was induced by cytokines following a rapid decline in B lymphocytes. The induction of Otud-6b is probably due to robust response after serum starvation and cytokine stimulation, which is a less physiological condition. However, the rapid down-regulation of Otud-6b is probably required to maintain a normal cell cycle as enforced Otud-6b expression can cause cell cycle arrest in Ba/F3 cells. What is more, Otud-6b is not expressed (or not detectable) in Ba/F3 cells continually growing in 1 ng/ml IL-3 (**Figure 1B**). Taken together, Otud-6b probably participates in B lymphocytes cell cycle as a negative regulator. Although these characteristics of Otud-6b are identified, there are still many questions need further study.

### Cytokine-inducible enhancer and transcription factors

Otud-6b induction-decline expression pattern is similar to those of mouse DUB/USP17 family member Dub-1 and Dub-2 [5], [14], which have cytokine-inducible enhancer in the promoter regions, such as AP-1 and ETS TF binding sites [13], [30]. Although the enhancer element of Otud-6b from −1515 bp to −1397 bp of Otud-6b is structurally different from those of Dub-1, Dub-1a, and Dub-2a, the functional transcription factor binding sites are very similar as the conserved ETS binding site is required for Otud-6b enhancer activity as well (**Figure S9**). However, a number of transcription factors could bind to the ETS binding site, and which ones are required for Otud-6b induction need further investigation.

### Regulation of OTUD-6B mRNA stability

Degradation of mRNA is important in the regulation of gene expression [31]. MicroRNAs are post-transcriptional regulators that bind to complementary sequences in the 3′UTR of target mRNAs, usually resulting in mRNA degradation and gene silencing [25]. Besides microRNAs, short-lived transcripts can also be controlled through RNA-binding proteins [32], such as TTP, AUF1, and BRF1, which can bind to the AREs in 3′-UTR of many cytokine and proto-oncogene mRNAs [27]. On the other hand, HuR, a ubiquitously expressed member of the Hu family, could stabilize ARE containing transcripts [33]. In this study we found that TTP could regulate the post-transcriptional level of Otud-6b mRNA through the 3′-UTR and the AU-rich sequences in the 3′-UTR play vital roles in contributing the stability of Otud-6b mRNA. However, the mechanism of TTP mediated Otud-6b mRNA degradation is still unclear. TTP has been reported to bind to varieties of AU-rich sequences in the 3′-UTR of short-lived transcripts, including TNF-alpha [27], GM-CSF [34], and c-fos [35]. Many of these TTP binding sites are functional AREs with tandemly repeat copies of the AUUUA motif. Few exceptions have been reported such as MHC class I, which contains non-AU-rich element [29]. Although we identified TTP binds to Otud-6b mRNA and the AU-rich sequences in the 3′UTR 2721–2950 region may contain the AU-rich element (ARE), which motifs (AREs or non-AREs) in the Otud-6b mRNA are responsible for TTP binding is unclear. Since TTP has some flexibility in its sequence requirements for binding, future detailed mutagenesis in the 3′-UTR of Otud-6b mRNA, especially the AU-rich sequences in the 3′UTR 2721–2950 regions, would be necessary to define the precise sequence requirements for TTP binding. On the other hand, TTP knock-down could not completely block the degradation of Otud-6b mRNA, other possible mechanisms involved in Otud-6b mRNA regulation will need further careful examination.

### OTUD-6B substrate(s) and cell cycle

Enforced expression of OTUD-6B causes Ba/F3 cell cycle arrest at G1 phase, while knockdown of Otud-6b showed no effect (**Figure S5**). During the G1-S transition, cyclin D type protein is synthesized in response to mitogenic stimulation and cyclin D-CDK4 complex can phosphorylate pRb which can induce cyclin E synthesis. When there is more cyclin E than p27, the excess cyclin E triggers phosporylation of p27, which is then started to be degraded [36], [37]. We re-checked cell cycle regulators in OTUD-6B overexpressed Ba/F3 cells and found that p27 level was up-regulated and cyclin E level was down-regulated (**Figure S10**), which correlates with cell cycle progression. However, the effect on cyclin D2 levels was modest; and how OTUD-6B cause such down-regulation still need further investigation as cyclin D2 is unlikely a substrate of OTUD-6B because we also did not observe any deubiquitinating activity of OTUD-6B on cyclin D2. Nevertheless, OTUD-6B probably could directly affect either the synthesis or the degradation process of cyclin D2 as overexpressing OTUD-6B in Hela cells could also cause cyclin D2 expression level down-regulation even without affecting cell cycle (**Figure S10**). Interestingly, we also did not observe any effect on p27 and cyclin E in Hela cells overexpressing OTUD-6B. The lack of cell cycle arresting effect in Hela cells is probably due to deregulated cell cycle in cancer cell line [38].

Therefore, it is important to further investigate which substrate(s) or pathway OTUD-6B regulates to control the level of cyclin D2. Many pathways have been reported to regulate cyclin D2 level in different tissues. For example, cyclin D2 is a direct target gene of Myc [39] and PU.1 transcription factor [40]. Its expression can also be induced by colony-stimulating factor-1 receptor through Src, MAPK/ERK kinase, and c-Myc pathways in macrophages [41]. On the other hand, GSK3 beta can suppress cyclin D2 expression by tumor suppressor PTEN [42]. Due to the complexity of cyclin D2 signaling pathway, we had not found the direct substrate of OTUD-6B yet. While preparing this article, we noticed that recent work reported by Mathew E. Sowa et al. used a CompPASS method in a global proteomic analysis on DUBs, including OTUD-6B, and their associated protein complexes [43]. However, likely because of the complexity of the substrate(s) and the transient nature of DUB-substrate interaction, they also failed to identify the right substrate(s) for OTUD-6B. However, further investigations on identifying OTUD-6B substrates will still be needed to understand the mechanism of cell cycle regulation by OTUD-6B.

## Materials and Methods

### Cell Lines and Cell Culture

Ba/F3 cells were cultured in RPMI1640 medium supplemented with 10% (v/v) FBS (Invitrogen) and 1 ng/ml recombinant mouse IL-3 (R&D systems) [5]. Recombinant mouse IL-2, IL-3, IL-4, IL-13, and granulocyte-macrophage colony-stimulating factor (GM-CSF) were purchased from R&D systems. Before addition of the cytokines for induction, Ba/F3 cells were cultured without serum and IL-3 for two hours. Hela cells were cultured in DMEM medium with 10% FBS. 293T cells were cultured in DMEM medium supplemented with 10% FBS. Raji cells were cultured in RPMI-1640 medium supplemented with 10% FBS. Ba/F3 cells were kindly supplied by prof. Xin yuan Liu (Shanghai Institute of Biochemistry and Cell Biology, SIBS, CAS). Hela cells, 293T cells, Raji cells, and CTLL2 cells were provided by the Cell Bank, Shanghai Institute of Biochemistry and Cell Biology, SIBS, CAS.

### Mouse Primary B Cells Isolation and Induction

Dynabeads® Mouse pan B (B220) (Invitrogen) can be used directly on whole blood samples from six 4-week C57BL/6 mice according to the manufacturer's procedures. Mouse B cells can then be lysed on the beads using DETACH BEAD CD19 (Invitrogen) to detach the cells from the beads. Isolated mouse B cells will be bead and antibody free and in perfect shape for downstream IL-3 induction.

### OTUD-6B Cloning and Semi-quantitative RT-PCR of Otud-6b

OTUD-6B cDNA was amplified from the total RNA of Raji cells by RT-PCR with specific primers. RT-PCR was performed at 94°C for 1 min, 50°C for 30 s, and 72°C for 3.5 min for a total of 40 cycles. The amplified cDNA was subcloned into the pGEMT-easy vector (Promega) and transformed into E.coli DH5α (Clontech). Total RNA was isolated by using Trizol (Invitrogen). Semi-quantitative RT-PCR was performed using a pair of specific primers for Otud-6b or Otud-6a (**Table S3**) at 94°C for 1 min, 50°C for 30 s, and 72°C for 1 min for a total of 35 cycles. For cell cycle regulators screens with RT-PCR, OTUD-6B, GAPDH, cyclin D1, cyclin D2, cyclin D3, p21, p27, p15, p16, cdk4, cdk6, cdc2, cyclin E, Rb, and c-Myc genes were performed with the specific primers (**Table S1**) at 94°C for 1 min, 55°C for 30 s, and 72°C for 30 s for a total of 30 cycles.

### Site-directed Mutagenesis

The OTUD-6B (C188S) mutant was generated by using a QuikChange TM site-directed mutagenesis kit (Stratagene) to replace Cys_188_ with Ser according to the manufacturer's instructions.

### Deubiquitination Assay

The deubiquitination assay has been previously described [5]. In brief, ubiquitin-β-galactosidase fusion protein (Ub-Met-β-gal) was expressed from a pACYC184-based plasmid in MC1061 cells. Purified GST, GST-OTUD-6B WT, GST-OTUD-6B C188S, GST-CYLD WT, and GST-CYLD CS fusion protein was incubated with Ub-Met-β-gal supernatant at 4°C with rotation for 4 hours, then the total protein was analyzed by immunoblot with anti-beta-gal antibodies (Sigma).

### Trypan Blue Staining

2×10^5^ Ba/F3 cells transfected with pcDNA3.1(+), OTUD-6B WT, and OTUD-6B C188S vectors and collected after different time (0 hour, 16 hours, 32 hours, and 48 hours), and then washed with 4°C PBS, and stained with 0.1 ml of 0.4% Trypan Blue for 5 min at room temperature. Then cells were observed and counted under a microscope.

### Cell Cycle Analysis and Apoptosis Assay

Ba/F3 cells were transfected with pcDNA3.1(+), OTUD-6B WT, and OTUD-6B C188S vectors. Thirty-two hours later, cells were washed with 4°C PBS and fixed with 70% ethanol at 4°C overnight. Then cells were treated with 200 µl RNase in 37°C water for 30 minutes and stained with propidium iodide (PI, Sigma). Cell cycle distribution was measured by flow cytometric assay, MODFIT LT of FACS Calibur (Becton Dickinson). PI-Annexin V assay was performed according to the manufacturer's instructions and analyzed with CELLQUEST PRO (Becton Dickinson).

### Tet-on Systems and Cell Cycle Regulators Screen

Doxycycline (DOX, Sigma)-inducible OTUD-6B expressing Hela cells were prepared as in Ref [44]. In brief, Hela cells stably transfected with pTet-On (Clontech) HA-OTUD-6B WT were treated with or without 2.5 µg/ml DOX. Total RNAs from both DOX (−) and DOX (+) Hela cells were collected and subjected to real-time PCR (**Table S2**) by an iCycler iQ thermal cycler system (Bio-Rad). Cycling parameters for real-time PCR were the same as mentioned before. Cell extracts were also collected and subjected for immunoblot with antibodies against OTUD-6B, c-Myc (Santacruz), GAPDH, cyclin D1, cyclin D2, cyclin D3, p21, p27, p15, p16, cdk4, cdk6, cdc2, cyclin E, and Rb antibodies (CST).

### Act.D Chase Assay for mRNA Stability Measurement

For mRNA stability measurement, Ba/F3 cells were incubated with 15 µg/ml actinomycin D (Act.D) to inhibit transcription. At the indicated time points after the addition of Act.D, cells were harvested and total RNA was extracted. The expression levels of Otud-6b at each time points were measured by qRT-PCR and normalized to the according G3pdh levels. The remaining mRNA was determined by comparison with the expression level of the Otud-6b at the zero time point (designated 1) when Act.D was added.

### Luciferase Assay

The pcDNA3.1, HA-TTP, HA-TTP Zn-FM constructs were generated as previously described [29]. The pGL3 luciferase constructs contain the Otud-6b 3′-UTR nucleotides (1221–2950, 1221–2720, and 2721–2950 of mouse Otud-6b cDNA). 293T cells were transfected with 1 µg of luciferase constructs and 500 ng of TTP expression constructs or pcDNA 3.1. Cells were lysed 24 h in 100 µl of luciferase lysis buffer (Promega), and 20 µl of each sample was read in a luminometer according to the manufacturer's protocol.

### Immunoprecipitation of mRNA-protein Complex

2×10^7^ Ba/F3 cells transfected with HA-TTP vector were used for the immunoprecipitation [45]. 24 hours later, Ba/F3 cells were stimulated with 10 pM IL-3 for 3 hours, inducing Otud-6b mRNA to high levels. The cells were then lysed for 10 min on ice in RNA immunoprecipitation (RIP) buffer [10 mM Tris-HCl at pH 7.6, 1.5 mM MgCl2, 100 mM NaCl, 1 mM DTT, 0.5% Nonidet P-40, 0.5% Triton X-100, 100 U/ml RNase inhibitor (Promega), 10 µL/mL protease inhibitor cocktails (Sigma)]. The cell lysate was centrifuged at 12000 g for 15 min at 4°C and the supernatant of the cytoplamic lysate was collected for RNA IP assay. Protein-RNA complexes were incubated with 1 ug anti-HA antibody and rotated for 12 hours at 4°C. Precleared pro-A Sepharose beads (Amersham) was used to pull-down anti-HA antibody or pre-immune serum by incubating overnight at 4°C with continuous rotation in RIP buffer. The beads were pelleted and the supernatant was removed. These beads and their bound complexes were recovered by centrifugation and washed six times with RIP buffer. Otud-6b mRNA was detected with RT-PCR using the specific primers.

### Statistical Analysis

Average values were expressed as mean ± SD. The statistical significance was assessed by Student's t test using SPSS 10.0 statistical software or Excel statistics. P<0.05 was considered statistically significant.

### Supplementary information associated experiments

OTUD-6B antibody production, OTUD-6B recombinant protein, Otud-6b enhancer luciferase plasmids, and Otud-6b siRNA design were shown in the (Materials and Methods S1).

## Supporting Information

Materials and Methods S1 Supplementary experimental materials and methods.
(0.04 MB DOC)

Figure S1 Amino acid alignment of human OTUD-6B and OTUD-6A, and mouse Otud-6b by CLUSTAL 2.0.12.
(0.30 MB TIF)

Figure S2 Immunoblot for GST-DUB fusion protein. Purified GST (lanes 1 and 4), GST-OTUD-6B WT (lane 2), GST-OTUD-6B C188S (lane 3), GST-CYLD WT (lane 5), and GST-CYLD CS (lane 6) were analyzed by immunoblot with an anti-GST monoclonal antibody.
(0.17 MB TIF)

Figure S3 IL-2 doesn't induce Otud-6b expression in Ba/F3 cells. A. Otud-6b RNA level in Ba/F3 cells after starvation and two hours stimulation with different concentrations (0, 0.01, 0.1, 1, 10, 100, and 1000 pM) of mouse IL-2. B. Otud-6b RNA level in Ba/F3 cells after starvation and stimulation with 10 pM mouse IL-2 for the indicated times (0, 0.5, 1, 2, 4, 8, and 16 hours).
(0.13 MB TIF)

Figure S4 Characterization of the rabbit antibody for endogenous OTUD-6B. A. Purified GST-OTUD-6A, GST-OTUD-6B WT, and GST-OTUD-6B C188S fusion protein was immunoblotted with anti-OTUD-6B antibody. Then, membranes were stripped and immunoblotted with anti-GST antibody. B. Ba/F3 cells were cultured with IL-3, then transfected with pcDNA3.1(+), HA-OTUD-6B WT, and HA-Otud-6b WT vectors. Immunoblot was performed with anti-OTUD-6B, anti-HA, and anti-GAPDH antibodies.
(0.24 MB TIF)

Figure S5 Knockdown of Otud-6b has no effect on cell growth. A&B. Ba/F3 cells were transfected with pSUPER mock, pSUPER Otud-6b siRNA-1 or 2 vectors, then stimulated with 10pM IL-3 for 2 hours after starvation. Total RNAs were analyzed by RT-PCR, and cell extracts were immunoblotted with anti-HA antibody. G3PDH was used as a loading control. C. pSUPER Mock and pSUPER Otud-6b siRNA-1 or 2 expressing Ba/F3 cells were seeded at 2×105 cells per ml and stimulated by 10 pM IL-3 and then analyzed by trypan blue exclusion assay 16 hours later.
(0.31 MB TIF)

Figure S6 Knockdown of OTUD-6B has no effect on cyclin D2 level. A. The levels of Cyclin D2 had no difference when OTUD-6B was knocked down in Hela cells. Hela cells transfected with pSUPER mock, pSUPER-OTUD-6B siRNA-1 or 2. Twenty four hours later, cell lysates were collected and then subjected to immunoblot with anti-OTUD-6B, anti-cyclin D2 antibodies. GAPDH was used as loading control. B. The levels of Cyclin D2 had no difference when Otud-6b was knocked down in Ba/F3 cells. Ba/F3 cells were stimulated with 10 pM IL-3 for 2 hours.
(0.45 MB TIF)

Figure S7 Knockdown of AUF1, BRF1 and Dicer1 has no effect on Otud-6b mRNA level. A, B, and C. Ba/F3 cells were transfected with pSUPER mock, pSUPER AUF1, BRF1, and Dicer1 siRNA vectors. AUF1, BRF1, and Dicer1 mRNAs were analyzed by RT-PCR. G3PDH was used as a loading control. D and E. Otud-6b RNA level in Ba/F3 cells transfected with pSUPER AUF1, BRF1, and Dicer1 siRNA vectors after starvation and stimulation with 10 pM mouse IL-3 for the indicated times (0, 0.5, 1, 2, 4, 8, 12, 16, and 24 hours). F. Ba/F3 cells were transfected with pSUPER Mock, AUF1, BRF1, and Dicer1 siRNA vectors. 24 hours later, cells were stimulated by 10 pM IL-3 and incubated with 15 ug/ml Act.D for the time indicated. The qRT-PCR was performed to detect Otud-6b mRNA remaining at each time point.
(0.70 MB TIF)

Figure S8 Scrambled vector has no effect on the Otud-6b mRNA rapid degradation after induction. A. pSUPER Scramble, pSUPER siTTP-1 or 2 and HA-TTP vectors co-expressing Ba/F3 cells were seeded at 2×105 cells per ml. Scrambled siRNA sequence is 5′- GAGGAGCCGACGCTTAATA-3′. Cell extracts were immunoblotted with anti-HA antibody. GFP was used as a loading control. B. Otud-6b RNA level in Ba/F3 cells transfected with pSUPER scramble, siTTP-1,2 vectors after starvation and stimulation with 10 pM mouse IL-3 for the indicated times (0, 0.5, 1, 2, 4, 8, 12, 16, and 24 hours).
(0.31 MB TIF)

Figure S9 The Otud-6b gene contains a cytokine-inducible enhancer element. A. Alignment of the cytokine-inducible enhancer regions of mouse Dub-1a, Dub-1, Dub-2a, and the promoter region of mouse Otud-6b. The putative enhancer region of mouse Otud-6b contains one ETS and one GATA TF binding site. Mutation sites in motifs are indicated with arrows. B. Luciferase activity was assayed in Ba/F3 cells that were transfected with the indicated constructs. The cells were starved and then treated with or without 10 pM mouse IL-3 stimulation. Luciferase assays were performed after 16 hours of treatment. All ratios are from three independent experiments. P value was calculated by student's t test.
(0.41 MB TIF)

Figure S10 P27 and cyclin E levels in Ba/F3 cells and Hela cells when overexpression of OTUD-6B WT and OTUD-6B CS. A and B. Immunoblot for p27 and cyclin E on Ba/F3 cells and Hela cells overexpressing pcDNA3.1(+), HA-OTUD-6B WT, and HA-OTUD-6B C188S. C. Cell cycle profile of PI-stained Hela cells when overexpressing pcDNA3.1(+), HA-OTUD-6B WT, and HA-OTUD-6B C188S.
(0.51 MB TIF)

Table S1 Primers used for RT-PCR in cell cycle regulators screen.
(0.04 MB DOC)

Table S2 Primers used for real time PCR in cell cycle regulators screen.
(0.04 MB DOC)

Table S3 Subcloning primers used in this study.
(0.05 MB DOC)
