# Supplementary material for: Evidence for OTUD-6B Participation in B Lymphocytes Cell Cycle after Cytokine Stimulation
Source: PLoS One. 2011 Jan 18;6(1):e14514. doi: 10.1371/journal.pone.0014514 (PMC3022568; doi:10.1371/journal.pone.0014514)
Supplement: Materials and Methods S1 — Supplementary experimental materials and methods. (0.04 MB DOC) [file pone.0014514.s001.doc]

**Supporting Information**

**Experimental materials and methods**

**Antibody production**

An OTUD-6B polyclonal antibody was generated by Abmart Inc. (Shanghai, China). Synthetic peptides (KNAVPKNDKKRRK, PRISKAQKRREKK, and RQLEIKQIPSDGHC) were used as antigens in immunization. Those peptides were designed specifically for OTUD-6B corresponding to OTUD-6B amino acids 64-76, 125-138, and 175-188. Two rabbits were injected with 600 µg antigen (200 µg of each peptide) in Freund's complete adjuvant (FCA) each time per rabbit for four times. The immunization period was 1/2/2/1 weeks for each rabbit. The antibodies were purified by affinity and the titer of the rabbit serum was tested by ELISA after the fourth immunization.

**Design of shRNA of both human OTUD-6B and mouse Otud-6b**

Human OTUD-6B shRNA and Mouse Otud-6b shRNA target sequence was designed according to ref [1]. Complementary oligonucleotides encoded a hairpin structure with a 19-mer stem derived from the mRNA target site. A 19-bp loop sequence separated the two complementary domains. The 5' end of two oligonucleotides was Bgl II and Hind III restriction site overhangs. Each pair of oligonucleotides was annealed and ligated into the Bgl II and Hind III sites of linearized pSUPER shRNA expression vector (Oligoengine).

**Recombinant protein**

A 972-bp ORF fragment from the wild-type OTUD-6B cDNA and a CDS containing a missense mutation (C188S) were generated by PCR and inserted in frame into pGEX-2TK (Pharmacia) downstream of the glutathione S-transferase (GST) coding element. GST, GST-OTUD-6B WT, GST-OTUD-6B C188S, GST-CYLD WT, and GST-CYLD CS fusion protein was expressed in BL21 *E.coli* cells (Stratagene). Cultures were grown at 30 °C to an OD600 of 0.5 and the temperature was lowered to 10 °C for 65h. Resulting pellets were resuspended and lysed in a buffer containing 50 mM HEPES-NaOH pH 8.0, 300 mM NaCl, 10% glycerol, 5 mM 2-mercaptoethanol, 5 mM imidazole, and 0.1% Brij35. The supernatant was then bound to Glutathione Sepharose 4B (Amersham), washed in the same buffer, and eluted with the elution buffer.

**Enhancer elements construction and luciferase transactivation experiment**

The putative enhancer element of Otud-6b from -1515 bp to -1397 bp of Otud-6b gene was amplified by PCR and subcloned into the pGL3 basic vector (Promega). Mutation of EST and GATA TF binding sites was performed by site-mutagenesis on the pGL3-Otud-6b WT enhancer vector [2]. Transient transfection of Ba/F3 cells and luciferase reporter gene assays were performed as previously described [2,3]. After electroporation (AMAXA), Ba/F3 cells were treated with or without 10 pM mouse IL-3 for 16 hours. Luciferase and renella levels were detected according to dual luciferase assay kit procedures (Promega).

**References**

1. Kayagaki N, Phung Q, Chan S, Chaudhari R, Quan C, et al. (2007) DUBA: a deubiquitinase that regulates type I interferon production. Science 318: 1628-1632.

2. Baek KH, Kim MS, Kim YS, Shin JM, Choi HK (2004) DUB-1A, a novel deubiquitinating enzyme subfamily member, is polyubiquitinated and cytokine-inducible in B-lymphocytes. J Biol Chem 279: 2368-2376.

3. Baek KH, Mondoux MA, Jaster R, Fire-Levin E, D'Andrea AD (2001) DUB-2A, a new member of the DUB subfamily of hematopoietic deubiquitinating enzymes. Blood 98: 636-642.
