# Supplementary figures and images for: Evidence for OTUD-6B Participation in B Lymphocytes Cell Cycle after Cytokine Stimulation
Source: PLoS One. 2011 Jan 18;6(1):e14514. doi: 10.1371/journal.pone.0014514 (PMC3022568; doi:10.1371/journal.pone.0014514)

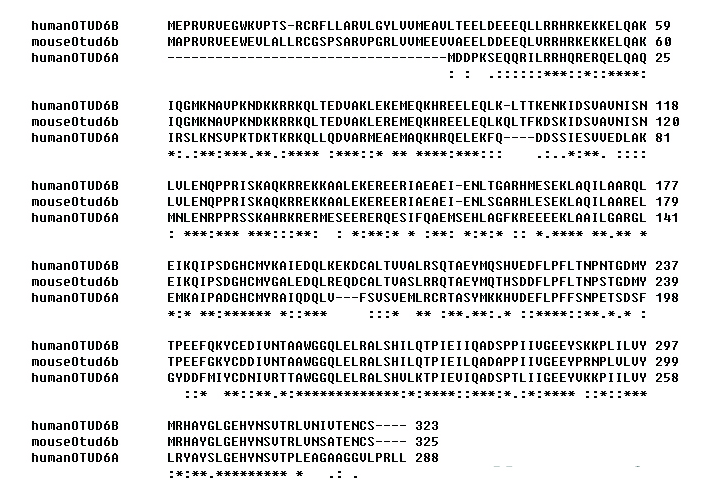

Supplement: Figure S1 — Amino acid alignment of human OTUD-6B and OTUD-6A, and mouse Otud-6b by CLUSTAL 2.0.12. (0.30 MB TIF) [file pone.0014514.s002.tif]

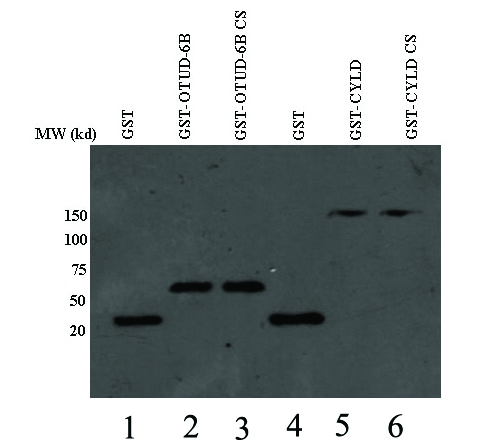

Supplement: Figure S2 — Immunoblot for GST-DUB fusion protein. Purified GST (lanes 1 and 4), GST-OTUD-6B WT (lane 2), GST-OTUD-6B C188S (lane 3), GST-CYLD WT (lane 5), and GST-CYLD CS (lane 6) were analyzed by immunoblot with an anti-GST monoclonal antibody. (0.17 MB TIF) [file pone.0014514.s003.tif]

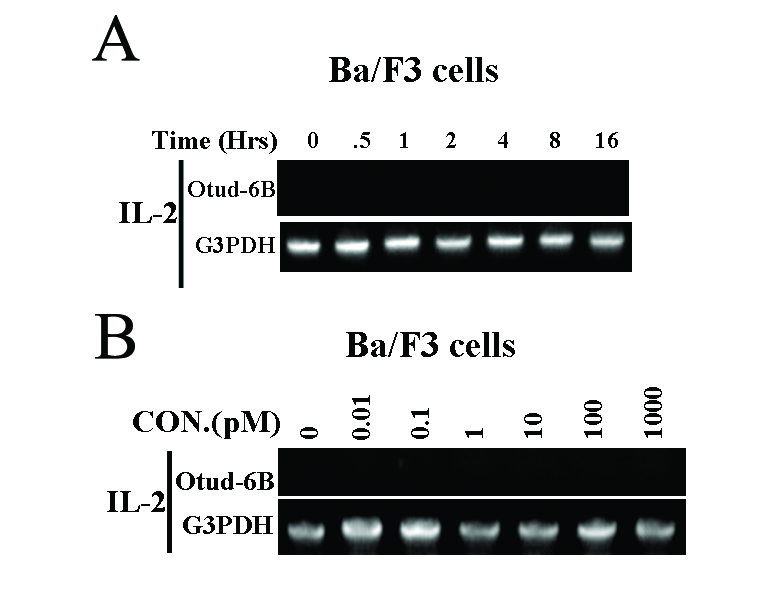

Supplement: Figure S3 — IL-2 doesn't induce Otud-6b expression in Ba/F3 cells. A. Otud-6b RNA level in Ba/F3 cells after starvation and two hours stimulation with different concentrations (0, 0.01, 0.1, 1, 10, 100, and 1000 pM) of mouse IL-2. B. Otud-6b RNA level in Ba/F3 cells after starvation and stimulation with 10 pM mouse IL-2 for the indicated times (0, 0.5, 1, 2, 4, 8, and 16 hours). (0.13 MB TIF) [file pone.0014514.s004.tif]

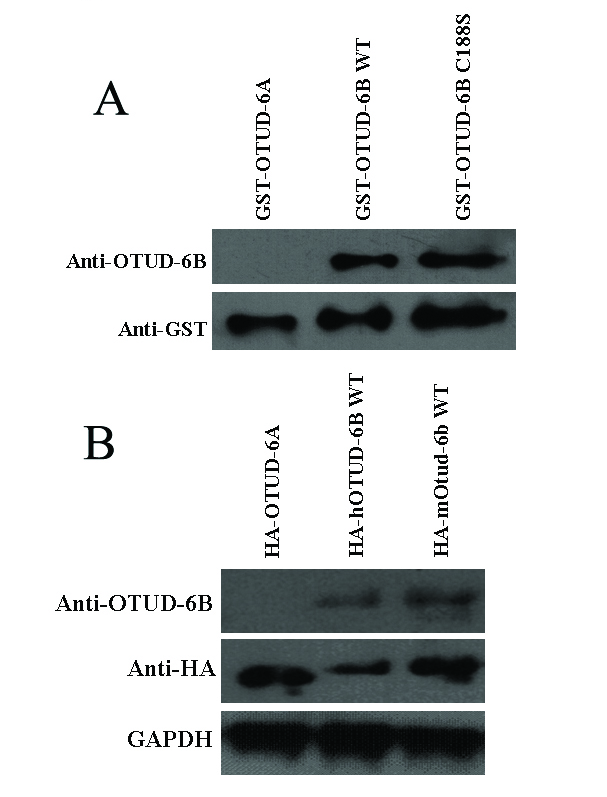

Supplement: Figure S4 — Characterization of the rabbit antibody for endogenous OTUD-6B. A. Purified GST-OTUD-6A, GST-OTUD-6B WT, and GST-OTUD-6B C188S fusion protein was immunoblotted with anti-OTUD-6B antibody. Then, membranes were stripped and immunoblotted with anti-GST antibody. B. Ba/F3 cells were cultured with IL-3, then transfected with pcDNA3.1(+), HA-OTUD-6B WT, and HA-Otud-6b WT vectors. Immunoblot was performed with anti-OTUD-6B, anti-HA, and anti-GAPDH antibodies. (0.24 MB TIF) [file pone.0014514.s005.tif]

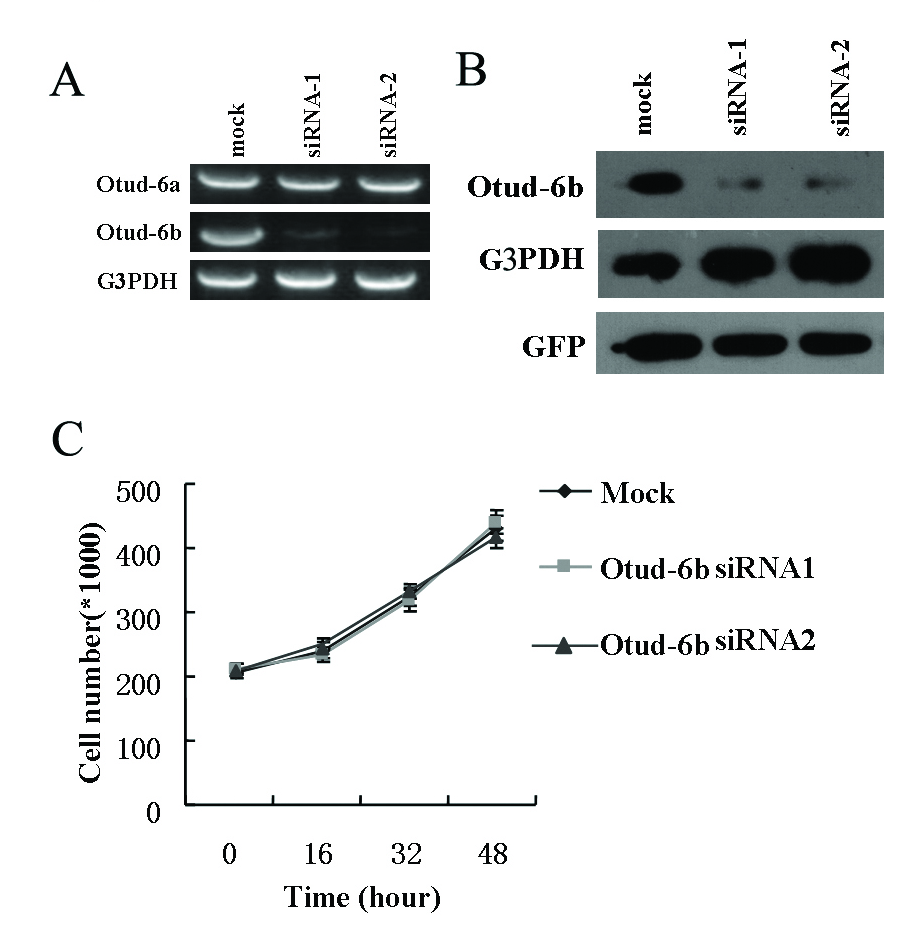

Supplement: Figure S5 — Knockdown of Otud-6b has no effect on cell growth. A&B. Ba/F3 cells were transfected with pSUPER mock, pSUPER Otud-6b siRNA-1 or 2 vectors, then stimulated with 10pM IL-3 for 2 hours after starvation. Total RNAs were analyzed by RT-PCR, and cell extracts were immunoblotted with anti-HA antibody. G3PDH was used as a loading control. C. pSUPER Mock and pSUPER Otud-6b siRNA-1 or 2 expressing Ba/F3 cells were seeded at 2×10^5^ cells per ml and stimulated by 10 pM IL-3 and then analyzed by trypan blue exclusion assay 16 hours later. (0.31 MB TIF) [file pone.0014514.s006.tif]

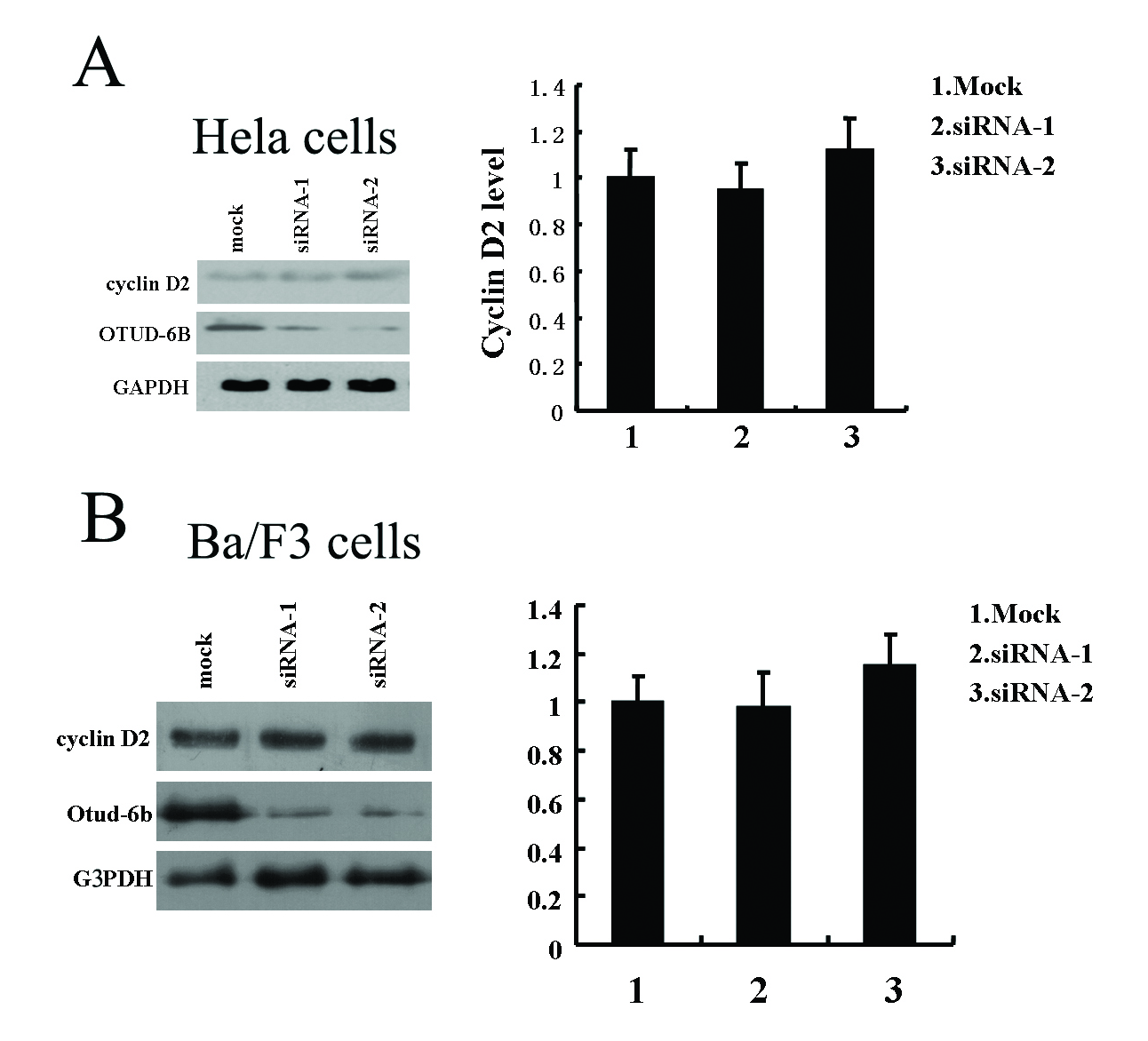

Supplement: Figure S6 — Knockdown of OTUD-6B has no effect on cyclin D2 level. A. The levels of Cyclin D2 had no difference when OTUD-6B was knocked down in Hela cells. Hela cells transfected with pSUPER mock, pSUPER-OTUD-6B siRNA-1 or 2. Twenty four hours later, cell lysates were collected and then subjected to immunoblot with anti-OTUD-6B, anti-cyclin D2 antibodies. GAPDH was used as loading control. B. The levels of Cyclin D2 had no difference when Otud-6b was knocked down in Ba/F3 cells. Ba/F3 cells were stimulated with 10 pM IL-3 for 2 hours. (0.45 MB TIF) [file pone.0014514.s007.tif]

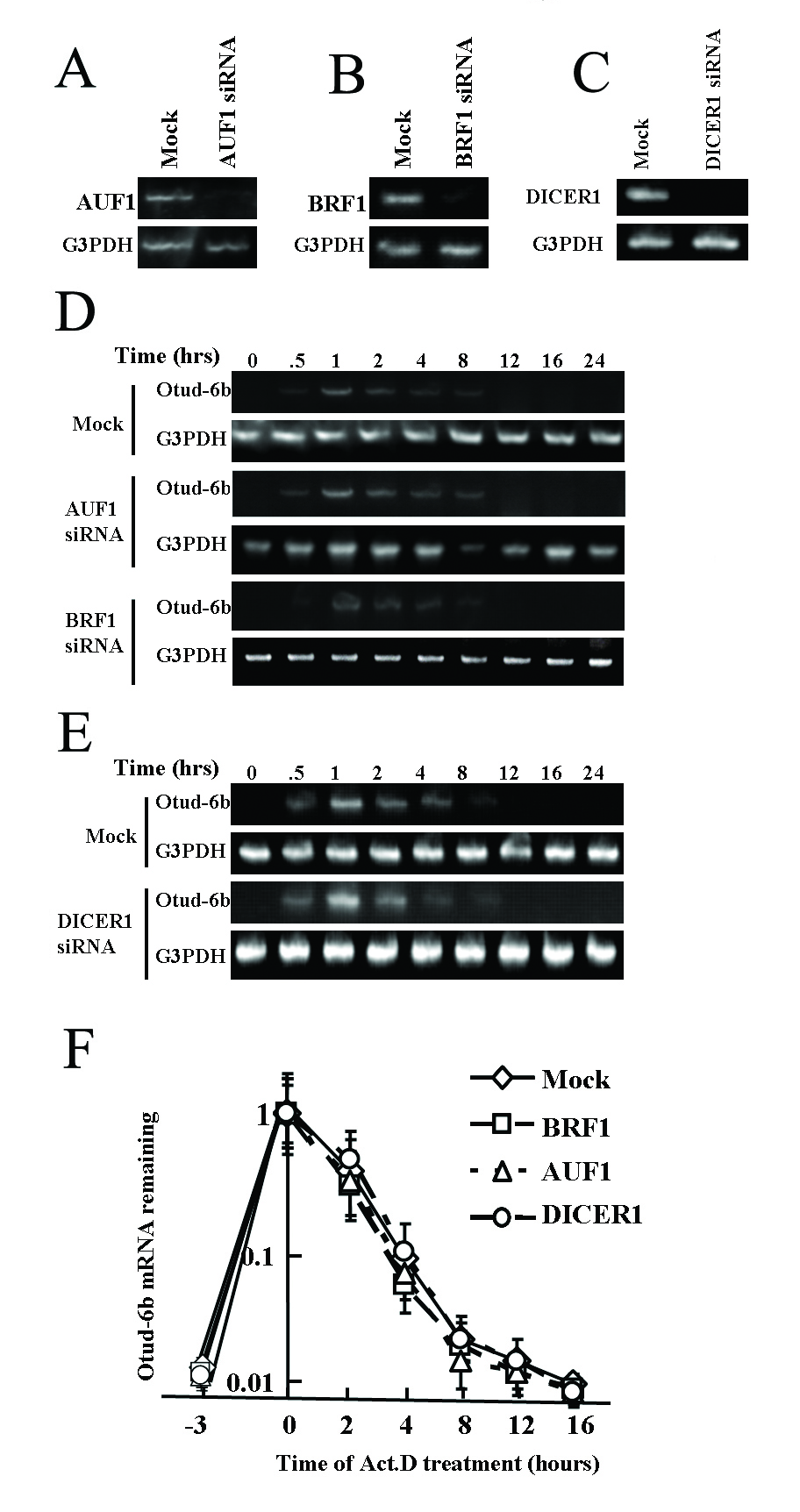

Supplement: Figure S7 — Knockdown of AUF1, BRF1 and Dicer1 has no effect on Otud-6b mRNA level. A, B, and C. Ba/F3 cells were transfected with pSUPER mock, pSUPER AUF1, BRF1, and Dicer1 siRNA vectors. AUF1, BRF1, and Dicer1 mRNAs were analyzed by RT-PCR. G3PDH was used as a loading control. D and E. Otud-6b RNA level in Ba/F3 cells transfected with pSUPER AUF1, BRF1, and Dicer1 siRNA vectors after starvation and stimulation with 10 pM mouse IL-3 for the indicated times (0, 0.5, 1, 2, 4, 8, 12, 16, and 24 hours). F. Ba/F3 cells were transfected with pSUPER Mock, AUF1, BRF1, and Dicer1 siRNA vectors. 24 hours later, cells were stimulated by 10 pM IL-3 and incubated with 15 ug/ml Act.D for the time indicated. The qRT-PCR was performed to detect Otud-6b mRNA remaining at each time point. (0.70 MB TIF) [file pone.0014514.s008.tif]

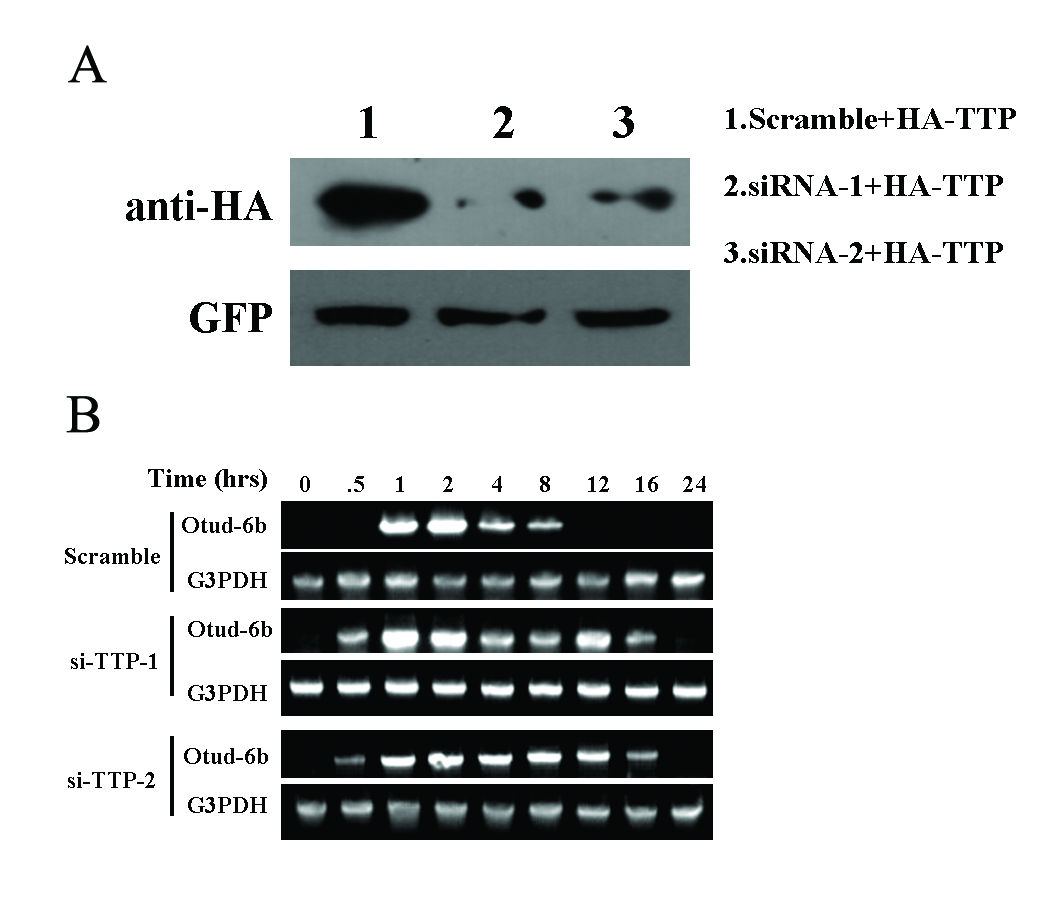

Supplement: Figure S8 — Scrambled vector has no effect on the Otud-6b mRNA rapid degradation after induction. A. pSUPER Scramble, pSUPER siTTP-1 or 2 and HA-TTP vectors co-expressing Ba/F3 cells were seeded at 2×10^5^ cells per ml. Scrambled siRNA sequence is 5′- GAGGAGCCGACGCTTAATA-3′. Cell extracts were immunoblotted with anti-HA antibody. GFP was used as a loading control. B. Otud-6b RNA level in Ba/F3 cells transfected with pSUPER scramble, siTTP-1,2 vectors after starvation and stimulation with 10 pM mouse IL-3 for the indicated times (0, 0.5, 1, 2, 4, 8, 12, 16, and 24 hours). (0.31 MB TIF) [file pone.0014514.s009.tif]

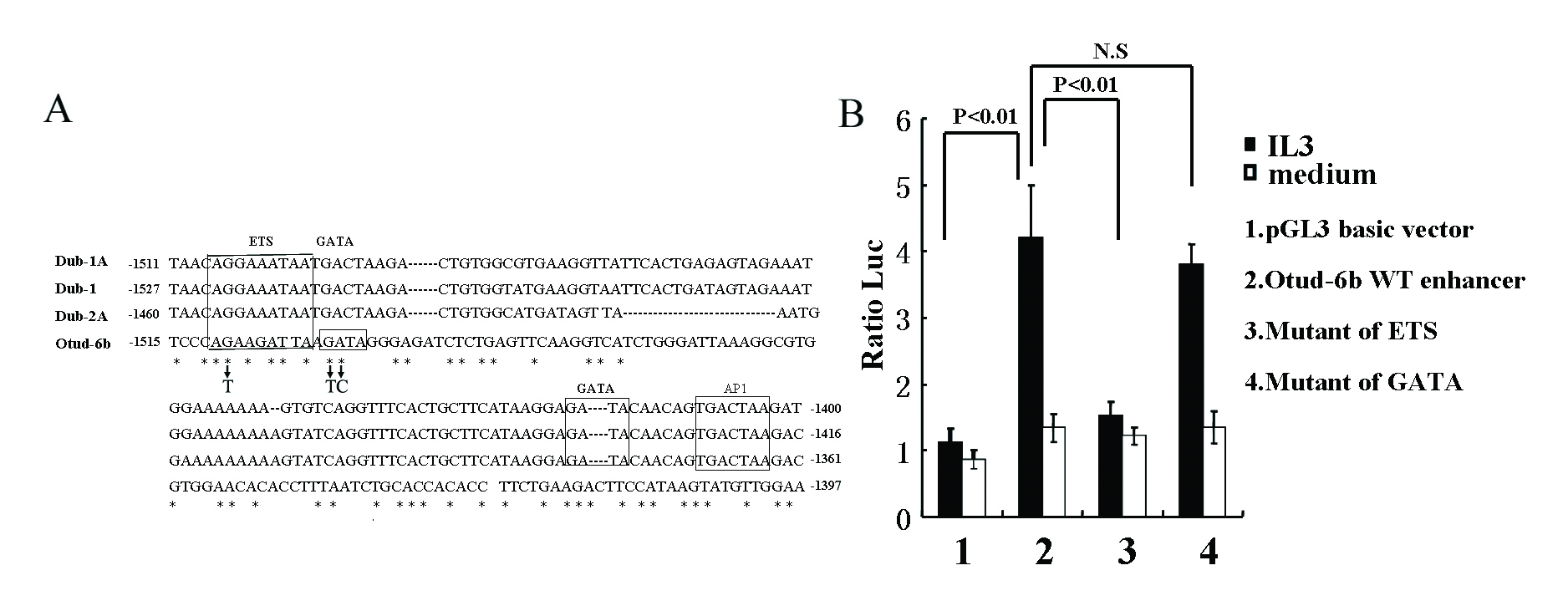

Supplement: Figure S9 — The Otud-6b gene contains a cytokine-inducible enhancer element. A. Alignment of the cytokine-inducible enhancer regions of mouse Dub-1a, Dub-1, Dub-2a, and the promoter region of mouse Otud-6b. The putative enhancer region of mouse Otud-6b contains one ETS and one GATA TF binding site. Mutation sites in motifs are indicated with arrows. B. Luciferase activity was assayed in Ba/F3 cells that were transfected with the indicated constructs. The cells were starved and then treated with or without 10 pM mouse IL-3 stimulation. Luciferase assays were performed after 16 hours of treatment. All ratios are from three independent experiments. P value was calculated by student's t test. (0.41 MB TIF) [file pone.0014514.s010.tif]

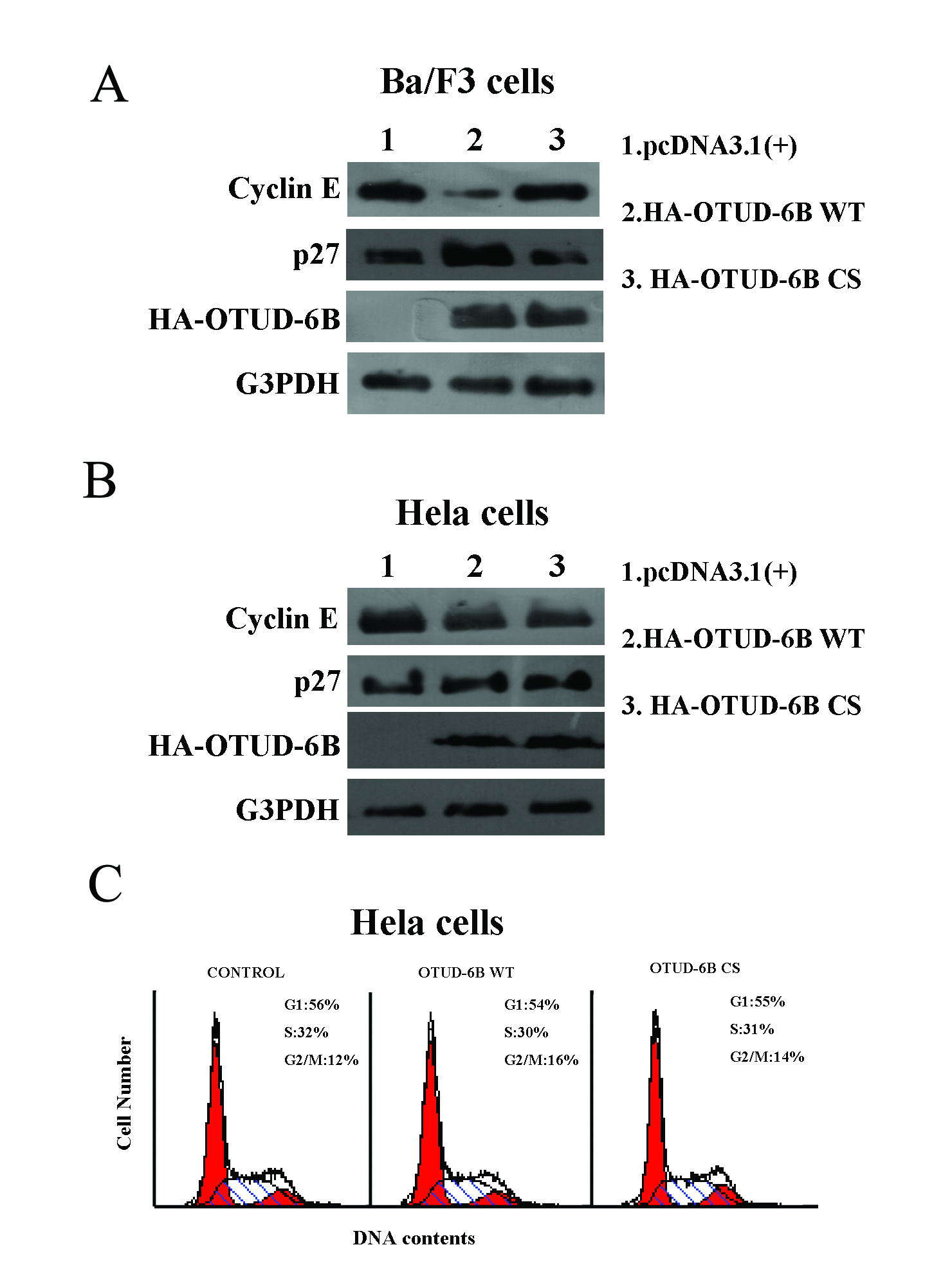

Supplement: Figure S10 — P27 and cyclin E levels in Ba/F3 cells and Hela cells when overexpression of OTUD-6B WT and OTUD-6B CS. A and B. Immunoblot for p27 and cyclin E on Ba/F3 cells and Hela cells overexpressing pcDNA3.1(+), HA-OTUD-6B WT, and HA-OTUD-6B C188S. C. Cell cycle profile of PI-stained Hela cells when overexpressing pcDNA3.1(+), HA-OTUD-6B WT, and HA-OTUD-6B C188S. (0.51 MB TIF) [file pone.0014514.s011.tif]
