# Supplementary material for: Evidence for OTUD-6B Participation in B Lymphocytes Cell Cycle after Cytokine Stimulation
Source: PLoS One. 2011 Jan 18;6(1):e14514. doi: 10.1371/journal.pone.0014514 (PMC3022568; doi:10.1371/journal.pone.0014514)
Supplement: Table S1 — Primers used for RT-PCR in cell cycle regulators screen. (0.04 MB DOC) [file pone.0014514.s012.doc]

**Supporting Information Table S1:** Primers used for RT-PCR in cell cycle regulators screen.

| name | Forward primers | Reverse primers |
| --- | --- | --- |
| OTUD-6B | 5’-CCATTATAGTTGGTGAAGAAT-3’ | 5’-CTTCTTCAAGACAAATTACATT-3’ |
| GAPDH | 5’-agaaggctggggctcatttg-3’ | 5’-aggggccatccacagtcttc -3’ |
| cyclin D1 | 5’-AAGCCTCAGCCCTCCCCAGCTGCCAG-3’ | 5’-AACCAACAACAAGGAGGATG-3’ |
| cyclin D2 | 5'-CATGGAGCTGCTGTGCCACG-3' | 5'-CCGACCTACCTCCAGCATCC-3' |
| cyclin D3 | 5'-GCCGGGACCTGGCTGCTGTGATTGCAC-3' | 5'-AGAGGGCCTCTCCAGGGCTACAGG-3' |
| p21 | 5'-TTGATTAGCAGCGGAACA-3' | 5'-TACAGTCTAGGTGGAGAAACG-3' |
| p27 | 5'-CAA GTA CGA GTG GCA AGA-3' | 5'-TCC ATG AAG TCA GCG ATA-3' |
| p15 | 5'-CCAGAAGCAATCCAGGCGCG-3' | 5'-CGTTGGCAGCCTTCATCG-3' |
| p16 | 5’-CCCGCTTTCGTAGTTTTCAT-3’ | 5’-TTATTTGAGCTTTGGTTCTG-3’ |
| cdk4 | 5’-ATGGCTGCCACTCGATATGAACCC-3’ | 5’-GTACCAGAGCGTAACCACCACAGG-3’ |
| cdk6 | 5’-CCGAGTAGTGCATCGCGATCTAA-3’ | 5’-CTTTGCCTAGTTCATCGATATC-3’ |
| cdc2 | 5'-GGTTCCTAGTACTGCAATTCG-3' | 5'-TTTGCCAGAAATTCGTTTGG-3' |
| cyclin E | 5'-CTGGATGTTGACTGCCTTGA-3' | 5'-CCGCTGCTCTGCTTCTTAC-3’ |
| Rb | 5'-agtatgactcatgagagacaggcatttgg-3' | 5'-gtgaggtattggtgacaaggtagggggcct-3' |
| c-Myc | 5’-TACCCTCTCAACGACAGCAG-3’ | 5’-TCTTGACATTCTCCTCGGTG-3’ |
