# Supplementary material for: Evidence for OTUD-6B Participation in B Lymphocytes Cell Cycle after Cytokine Stimulation
Source: PLoS One. 2011 Jan 18;6(1):e14514. doi: 10.1371/journal.pone.0014514 (PMC3022568; doi:10.1371/journal.pone.0014514)
Supplement: Table S2 — Primers used for real time PCR in cell cycle regulators screen. (0.04 MB DOC) [file pone.0014514.s013.doc]

**Supporting Information Table S2:** Primers used for real time PCR in cell cycle regulators screen.

| name | Forward primers | Reverse primers |
| --- | --- | --- |
| OTUD-6B | 5’-GAGCTTGATGAGGAAGAGCAGCT-3’ | 5’-attcttgggaacagcgat-3’ |
| GAPDH | 5’-ccactcctccacctttgac-3’ | 5’-accctgttgctgtagcca-3’ |
| cyclin D1 | 5’-CTGGCCATGAACTACCTGGA-3’ | 5’-GTCACACTTGATCACTCTGG-3’ |
| cyclin D2 | 5’-CTGTGTGCCACCGACTTTAAGTT-3’ | 5’-GATGGCTGCTCCCACACTTC-3’ |
| cyclin D3 | 5’-CTGGCCATGAACTACCTGGA-3’ | 5’-CCAGGAAATCATGTGCAATC-3’ |
| p21 | 5’-gcgatggaacttcgactttgt-3’ | 5’-gggcttcctcttggagaagat-3’ |
| p27 | 5’-GGAGCAATGCGCAGGAATAA-3’ | 5’-TGGGGAACCGTCTGAAACAT-3’ |
| p15 | 5’-AGAACAAGGGCATGCCCAGT-3’ | 5’-ATCATCATGACCTGGATCGC-3’ |
| p16 | 5’-CGCAACCGCCGAACG-3’ | 5’-TTTTTTCGTTAGTATCGGAGGAAGA-3’ |
| cdk4 | 5’-TTGCATCGTTCACCGAGATC-3’ | 5’-CTGGTAGCTGTAGATTCTGGCCA-3’ |
| cdk6 | 5’-AGAAGAAGACTGGCCTAGAG-3’ | 5’-TGGAAGTATGGGTGAGACAGG-3’ |
| cdc2 | 5’-ggttcctagtactgcaattcg-3’ | 5’-tttgccagaaattcgtttgg-3’ |
| cyclin E | 5’-TTCTTGAGCAACACCCTCTTCTGCAGCC-3’ | 5’-TCGCCATATACCGGTCAAAGAAATCTTGTGCC-3’ |
| Rb | 5’-GCTAGCCTATCTCCGGCTAAA-3’ | 5’-CTGGAAAAGGGTCCAGATGA-3’ |
| c-Myc | 5’-GCCACGTCTCCACACATCAG-3’ | 5’-TCTTGGCAGCAGGATAGTCCTT-3’ |
| β-actin | 5’-accgagcgcggctacag-3’ | 5’-cttaatgtcacgcacgatttcc-3’ |
