# Supplementary material for: Evidence for OTUD-6B Participation in B Lymphocytes Cell Cycle after Cytokine Stimulation
Source: PLoS One. 2011 Jan 18;6(1):e14514. doi: 10.1371/journal.pone.0014514 (PMC3022568; doi:10.1371/journal.pone.0014514)
Supplement: Table S3 — Subcloning primers used in this study. (0.05 MB DOC) [file pone.0014514.s014.doc]

**Supporting Information Table S3:** Sub-cloning primers used in this study.

| name | Forward primers | Reverse primers |
| --- | --- | --- |
| Otud-6b RT-PCR | 5’-GAAAAGAAGGCTGCATTGGA-3’ | 5’-GTGTTCACAATATCGTCACA -3’ |
| Genomic Otud-6b PCR | 5’-agttagctttcttcaccaca-3’ | 5’-atattctgctcatggtcttg -3’ |
| Otud-6a RT-PCR | 5’-atgtctgacaccgagcaggaact -3’ | 5’-CCTCCTCCTCACGACGGTTG -3’ |
| cDNA of human OTUD-6B | 5’-gcgcacgcgcagcaccccattt -3’ | 5’-ACATCACATAATATAAGCAAAACTAT -3’ |
| OTUD-6B (C188S) mutant | 5'-tccatctgatggccacagtatgtataaagccatt -3' | 5'-aatggctttatacatactgtggccatcagatgga -3' |
| Enhancer region of Otud-6b (-1515~-1397bp) | 5'-TCCCAGAAGATTAAGATAG-3' | 5'-TTCCAACATACTTATGGAAGTCT-3' |
| Mutant ETS site | 5'-GTGGCTATGAATCCCATAAGATTAAGATAGGGA-3' | 5'-TCCCTATCTTAATCTTATGGGATTCATAGCCAC-3' |
| Mutant GATA site | 5'-GAATCCCAGAAGATTAATCTAGGGAGATCTCTGAGTTC-3' | 5'-GAACTCAGAGATCTCCCTAGATTAATCTTCTGGGATTC-3' |
| Human OTUD-6B shRNA-1 | 5’-gatcccccGATAATACAGGCAGATTCTTTtcaagagaaagaatctgcctgtattatcttttt -3’ | 5’-agctaaaaaGATAATACAGGCAGATTCTTTctcttgaaaagaatctgcctgtattatcgggg -3’ |
| Human OTUD-6B shRNA-2 | 5’-gatcccccCTAGACAGTTAGAAATTAATTtcaagagaattaatttctaactgtctagttttt -3’ | 5’-agctaaaaaCTAGACAGTTAGAAATTAATTctcttgaaattaatttctaactgtctaggggg -3’ |
| Mouse Otud-6b shRNA-1 | 5'-gatcccccAAGAACACAGCTACCTTACACtttcaagagaagtgtaaggtagctgtgttcttttttt -3' | 5'-agctaaaaaAAGAACACAGCTACCTTCACttctcttgaaagtgtaaggtagctgtgttcttgggg -3' |
| Mouse Otud-6b shRNA-2 | 5'-gatcccccAACACAGCTACCTTACACGAGtttcaagagaactcgtgtaaggtagctgtgttttttt -3' | 5'-agctaaaaaAACACAGCTACCTTACACGAGttctcttgaaactcgtgtaaggtagctgtgttgggg -3’ |
| Mouse TTP shRNA-1 | 5'-gatccccctCGCCACCCCAAGtACAAAtttcaagagaatttgtacttggggtggcgattttt-3' | 5'-agctaaaaatCGCCACCCCAAGtACAAAttctcttgaaatttgtacttggggtggcgagggg-3' |
| Mouse TTP shRNA-2 | 5'-gatcccccCtCtGCCACAAGttCtACCtttcaagagaaggtagaacttgtggcagagttttt-3' | 5'-agctaaaaaCtCtGCCACAAGttCtACCttctcttgaaaggtagaacttgtggcagaggggg-3' |
| Mouse BRF1 shRNA | 5’-GATCCTGTCCGAATCCCCTCACATGTTCAAGAGACATGTGAGGGGATTCGGACTTTTT-3’ | 5’-AGCTTAAAAAGTCCGAATCCCCTCACATGTCTCTTGAACATGTGAGGGGATTCGGGG-3’ |
| Mouse AUF1 shRNA | 5'-gatcccccAGAGGGGAGTATATCGGTTtcaagagAACCGATATACTCCCCTCT ttttt-3' | 5'-agctaaaaaAGAGGGGAGTATATCGGTTctcttgaAACCGATATACTCCCCTCTgggg-3' |
| Mouse Dicer1 shRNA | 5’-GATCCCCCTGCCTCACTTGACCTGAATTCAAGAGATTCAGGTCAAGTGAGGCAGTTTTT-3’ | 5’-agctaaaaaTGCCTCACTTGACCTGAATctcttgaATTCAGGTCAAGTGAGGCAgggg-3’ |
| Otud-6b 3′-UTR 1221-2950 luciferase | 5’-aaaaatgaagatttcaattta-3’ | 5’-TTTTTACAGGCTATAAATATATGTA-3’ |
| Otud-6b 3′-UTR 1221-2720 luciferase | 5’-aaaaatgaagatttcaattta-3’ | 5’-TGGCCATTTTAAAATAAAACAT-3’ |
| Otud-6b 3′-UTR 2721-2950 luciferase | 5’ -gtatatttatgaataaaatgt-3’ | 5’-TTTTTACAGGCTATAAATATATGTA-3’ |
